# Effects of Benzo[a]pyrene-DNA adducts, dietary vitamins, folate, and carotene intakes on preterm birth: a nested case–control study from the birth cohort in China

**DOI:** 10.1186/s12940-022-00859-7

**Published:** 2022-05-06

**Authors:** Nan Zhao, Weiwei Wu, Shiwei Cui, Haibin Li, Yongliang Feng, Ling Guo, Yawei Zhang, Suping Wang

**Affiliations:** 1 Medical Research Center/State Key Laboratory of Complex Severe and Rare Diseases, Peking Union Medical College Hospital, Chinese Academy of Medical Sciences and Peking Union Medical College, Beijing, China; 2 Department of Epidemiology, School of Public Health, Shanxi Medical University, Taiyuan, Shanxi China; 3 Chinese Center for Disease Control and Prevention, National Institute for Occupational Health and Poison Control, Beijing, China; 4 Institute of Urban Safety and Environmental Science, Beijing Academy of Science and Technology, Beijing, China; 5 National Cancer Center/National Clinical Research Center for Cancer/Cancer Hospital, Chinese Academy of Medical Sciences and Peking Union Medical College, Beijing, China

**Keywords:** Benzo[a]pyrene, DNA Adducts, Vitamins, Folate, Carotene, Preterm birth

## Abstract

**Background:**

Polycyclic aromatic hydrocarbons (PAHs) and its DNA adducts has been suggested to increase the risk of preterm birth (PB). Yet, few studies have been conducted to investigate this association, and the role of dietary nutrients intakes including vitamins, folate, and carotene during pre- and post-conception on this association has not been studied.

**Methods:**

Building upon a birth cohort in Taiyuan China, we conducted a nested case control study including 83 PB and 82 term births. Benzo[a]pyrene (BaP)-DNA adducts were measured by an improved LC-MC/MC analytic method. Dietary nutrient intakes were estimated from food frequency questionnaire using the Chinese Standard Tables of Food Consumption. Multivariable logistic regression model was used to examine the associations.

**Results:**

Increased risk of PB was observed as per interquartile increase in maternal BaP-DNA adduct level (OR = 1.27, 95%CI 0.95–1.67). Compared to low level (below mean) of maternal adducts, high level (above mean) of adducts was associated with the risk of PB (OR = 2.05, 95%CI 1.05–4.01). After stratified by dietary nutrients intakes, high adducts levels were associated with approximately 2–fourfold times increases in risk of PB among women with low vitamin A, C, E, folate, and carotene intakes during pre- and/or post-conception. Stronger stratified associations were consistently seen during preconception. Similar patterns were observed after additional adjustment for supplementation.

**Conclusions:**

Our study supports the hypothesis that high level of maternal PAHs exposure was significantly associated with increased risk of PB, and provides the first evidence that dietary vitamins, carotene, and folate intake levels may modify this association during different pregnancy windows. Our findings are relevant to identify recommendation for environment management and prenatal nutrition regarding pregnant women and newborns. Further investigation in other populations is warranted.

**Supplementary Information:**

The online version contains supplementary material available at 10.1186/s12940-022-00859-7.

## Introduction

Preterm birth (PB, < 37 completed gestational weeks) is the leading cause of neonatal morbidity and mortality worldwide and is associated with various infant morbidities and adult chronic diseases. Approximately 15 million PB infants are born each year worldwide [1–3], and the numbers continue to increase [4]; among about 3.3 million newborns die worldwide and nearly 27% are directly attributable to PB. As PB continues to emerge as a major public health concern, a solution to this growing problem is a priority for Millennium Development Goal 4 by the World Health Organization (WHO) [2]. PB is a complex and multifactorial outcome with etiologically distinct subtypes, few risk factors have been established.

Growing evidence supports environmental pollution has been linked to reproductive health [5–8]. Polycyclic aromatic hydrocarbons (PAHs) are ubiquitous environmental contaminants produced from combustion products of cigarette smoking, fossil fuels, industrial or domestic coal, and during cooking foods [9]. Recent epidemiological studies suggested prenatal exposure to ambient PAHs may be associated with adverse birth outcomes, including PB, low birth weight, intrauterine growth retardation, and childhood neurodevelopment [7, 9–14]. As widely used as a representative marker of exposure to total carcinogenic PAHs, Benzo[a]pyrene (BaP) has been identified as human mutagen, carcinogen, and endocrine disruptor, and is listed 8^th^ as the national priority list of hazardous substances by the Agency for Toxic Substances and Disease Registry [15, 16]. BaP is systematically distributed to form DNA adducts in numerous tissues after exposure to PAHs, the detectable adduct levels and the significant positive correlations between maternal and fetal adduct levels showed that genotoxic exposures can cross the human placenta to fetus [17]. BaP can induce DNA adducts in the fetus from the beginning of gestation [18], and have an estimated 3–4 months half-life in the body [19], therefore, it measures the effects of exposure, absorption, activation, detoxification, and repair within the fetus throughout pregnancy [20].

To date, only one study indirectly investigated BaP-DNA adducts levels, as a molecular dosimeter of PAHs exposure, associated with the risk of PB [12]. In addition, the role of dietary factors hypothesized to influence metabolism of BaP has not been considered. Antioxidant nutrients such as vitamins and carotene have been reported to reduce DNA damage related to PAHs exposure by inducing the activity of detoxifying enzymes and therefore help to protect against adverse health outcomes related to exposure to such contaminants [21, 22]. Folate plays an essential role in DNA synthesis, repair, and methylation [23]. It is particularly important in pregnancy and infancy, which involve rapid cell division and growth [24]. To the best knowledge, Duarte-Salles et al. have explored the effect of dietary vitamins and carotene that may modify the association between dietary BaP intake and PB, and found BaP intakes were associated with significant reductions in birth weight and length among women with low intakes of vitamin C [21]. Abramovici et al. reported vitamin C/E supplementation might associated with PB among smokers [25]. However, no study has examined the association between maternal BaP-DNA adducts, dietary intake of vitamins, folate and carotene, and risk of PB.

China has one fifth of the world population, and also the second largest number of PB with PB rates ranging from 4.1% to 18.9% [3, 4]. In light of very few studies conducted to investigate the relationship between maternal BaP-DNA adducts and PB risk, especially in Chinese population, and the literature gap regarding the effects of dietary vitamins, folate, and carotene on this association, we conducted a nested case–control study building upon a birth cohort in Taiyuan, China to examine the association between maternal BaP-DNA adduct, dietary intakes, and risk of PB with an improved LC–MS/MS detection method of BaP-DNA adduct and the Chinese Standard Tables of Food Consumption [26] for dietary nutrients estimation.

## Material and methods

### Study population

Details of the study population were described in previous publications [27, 28]. In brief, a birth cohort was established at the Department of Obstetrics and Gynecology of the First Affiliated Hospital of Shanxi Medical University in Taiyuan, China between March 2012 and November 2013. All the study procedures were approved by the Human Investigation Committee at Shanxi Medical University. All experiments were performed in accordance with relevant guidelines and regulations. Pregnant women who came to the hospital for delivery with gestational age ≥ 20 weeks, who had no mental illness, and who were aged 18 years or older were considered to be eligible. A total of 4,208 eligible women were contacted for participation and informed of study procedures upon their arrival at the hospital for delivery. An informed consent was obtained from all subjects, an in-person interview was conducted at the hospital by trained study interviewers using a standardized and structured questionnaire. The questionnaire collected data regarding demographic factors, reproductive and medical histories, behavioral factors, occupational and residential histories, physical activity, and diet. Information on both birth outcomes and pregnancy complications were abstracted from medical records. 523 women refused to participate and 26 did not complete in-person interviews, yielding 3,659 women (87% of eligible women contacted) who completed in-person interviews and 3,166 women (75%) who donated blood samples.

Building upon this birth cohort, we conducted a nested case–control study of BaP exposure and preterm birth. PB was defined as delivery prior to 37 completed weeks of gestation [1]. Gestational age at delivery was calculated in completed weeks from the first day of the last menstrual period. Term birth (control) was defined as delivery at 37 or more completed weeks of gestation. Pregnant women who had maternal blood samples available, who gave singleton live births without birth defects, and who had no chronic hypertension or cardiovascular diseases were included in our study. The estimated sample size with 0.80 power to detect the association of OR = 2.0 was 108 for each group. Of 110 PB randomly selected in this population who met the above criterion, we also excluded medically indicated PB (*N* = 27) in order to investigate the association between BaP exposure and PB without considering the effect of maternal complications, thus the final sample size was 83 cases and 83 controls (term births) from the same population who were frequency matched to the cases by age (± 1 years), residence, and season of conception (spring: March to May, summer: June to August, autumn: September to November, winter: December to February).

### DNA extraction and BaP-DNA adducts assays

Maternal whole blood was collected on the first day after delivery and was separated and stored immediately at -80 °C. DNA was extracted, isolated, and purified from whole blood samples according to a standard phenol–chloroform extraction method. Benzo[a]pyrenediol epoxide-dG (BPDE-dG) standards was synthesized according to the protocol reported by Beland et al. and described previously by Guo et al.[29]. The improved LC–MS/MS method was used to quantify BPDE-dG in human blood samples [29] with the limit of detection of BPDE-dG was 2.7 BPDE-dG/109dG, it was of high sensitivity and was with good repeatability and suitable for sample analysis of population-based study. This improved method was firstly performed in detection of BaP-DNA adducts by Guo et al. and validated to provide accurate results with small amount of human blood DNA (as low as ~ 10 µg) compared to the high-performance liquid chromatography (HPLC)/fluorescence method with ~ 100 µg of DNA used in previous studies [12, 14]. In brief, whole blood cell DNA (10 µg) was added 10 µL DNase I (0.5 unit/mL) and 10 pg of [^15^N_5_] BPDE-dG as I.S. for correcting recovery of BPDE-dG adduct. After incubation, the mixture was added 10 µL phosphodiesterase I (0.0002 unit/mL) and 10 µL alkaline phosphatase (0.004 unit/mL). The mixture was extracted by adding 500 µL of water-saturated n-butanol three times after another incubation. The nbutanol extracts were evaporated and dissolved in 50 µL of methanol and injected into LC–MS/MS for analysis.

Assays were performed on all samples that were of adequate quantity and quality for analysis. Of 166 subjects who had available whole blood samples, we excluded one subject with inadequate amount of DNA isolated (< 10 µg), which yield 165 adducts including 83 cases and 82 controls in the final analyses.

### Dietary intake and supplementation

Dietary information was collected via a semi-quantitative food frequency questionnaire. Daily dietary vitamin A, C, E (VA, VC, VE), folate, and carotene intake was estimated from the frequency of consumption and portion size of food items using the Chinese Standard Tables of Food Consumption [26] for each time period, this estimation has been used and validated by another birth cohort conducted in Lanzhou site [30, 31]. Information on vitamin or mineral supplements were asked for the following four time periods: pre-conception (12 months before pregnancy), first trimester (1–13 weeks), second trimester (14–27 weeks) and third trimester (> 27 weeks). For each time period, duration (how many weeks) and frequency (how many times per week) of supplementation were ascertained. Supplement users were defined as those who took supplement during pre-conception and/or pregnancy. Non-users were defined as those who never supplement during pre-conception and/or pregnancy.

### Statistical analysis

Chi-square tests were employed to compare the selected characteristics between PB and term births. Descriptive analyses of BaP-DNA adduct levels (µg/gDNA), dietary intake levels (mg, ug, or ug RE), and supplementation intake times among ever users between cases and controls were performed by using the Student’s t-test.

Multivariable unconditional logistic regression was used to examine the relationships between 1) maternal BaP-DNA adducts and risk of PB, 2) dietary vitamins, folate, carotene intakes and risk of PB by different pregnancy windows, and 3) maternal BaP-DNA adducts and risk of PB stratified by mean levels of dietary vitamins, folate, and carotene during preconception and entire pregnancy. Adduct levels were treated in the analyses both continuously as per quartile increase, and categorically as low vs. high (the mean level among controls was a cutoff point) maternal exposure. Dietary intake levels were considered as continuously in the students t-test, categorically in the association analysis, and two strata (≤ mean level, > mean level) in the stratified analysis. Potential confounding variables included maternal age (≤ 30, ≥ 30 years), education level (< college, ≥ college), family monthly income per capita (< 3000, ≥ 3000RMB), employment status during pregnancy (yes, no), pre-pregnancy body mass index (BMI) (≤ 18.5, 18.5–24, ≥ 24), parity (primiparous, multiparous), C-section (yes, no), newborn’s gender (female, male), passive smoking during pregnancy (yes, no), and activities during pregnancy (yes, no). Smoking during pregnancy was defined as women who smoked one or more cigarettes per day for at least one month (active), or women who were exposed to cigarette smoke at home, work, during social and recreational activities and/or while commuting to and from work for at least 30 min per week during pregnancy (passive); employment during pregnancy was defined as women who were ever employed during the entire pregnancy; activities during pregnancy was defined as women who had physical activities one year before and/or during pregnancy. No subject had alcohol consumption and active smoking during pregnancy, so they were not included in the final analyses. We additionally adjusted for supplementation status in the final models and compared the results. All analyses were performed using SAS software, version 9.4 (SAS Institute, Inc., Cary, North Carolina).

## Results

Distributions of selected characteristics for study population were presented in Table 1. Compared to women who delivered term births, women who delivered PB were more likely to have lower education level and family income, and fewer physical activities during pregnancy. Distributions of maternal age, employment status during pregnancy, pre-pregnancy BMI, parity, C-section, infant’s gender, passive smoking during pregnancy, and supplements intake were similar between term and PB groups.

**Table 1:** Distributions of selected characteristics between PB and term births

Characteristics	Term births (*N* = 82)		PBs (*N* = 83)		*P*^a^
	N	%	N	%	
Maternal age (years)					
< 30	52	63.4	54	65.1	0.83
≥ 30	30	36.6	29	34.9	
Highest education level					
< College	29	35.4	42	50.6	0.048
≥ College	53	64.6	41	49.4	
Family monthly income (RMB per capita)					
< 3,000	33	4.02	46	55.4	0.051
≥ 3,000	49	59.8	37	44.6	
Employment during pregnancy					
No	48	58.5	53	63.9	0.48
Yes	34	41.5	30	36.1	
Pre-pregnancy BMI^b^					
≤ 18.5	16	19.5	13	15.7	0.68
18.5–24.0	52	63.4	58	69.9	
≥ 24.0	14	17.1	12	14.5	
Parity					
Primiparous	57	69.5	51	61.5	0.28
Multiparous	25	30.5	32	38.6	
C-section					
No	51	62.2	41	49.4	0.098
Yes	31	37.8	42	50.6	
Gender					
Female	37	45.1	39	47.0	0.81
Male	45	54.9	44	53.0	
Passive smoking during pregnancy					
No	69	84.2	76	91.6	0.14
Yes	13	15.9	7	8.4	
Activities during pregnancy					
No	10	12.2	21	25.3	0.031
Yes	72	87.8	62	74.7	
Supplement intake					
Never	15	18.3	17	20.5	0.72
Ever	67	81.7	66	79.5	

^a^Calculated by Chi-square analysis

^b^Weight (kg) / height (m)^2^

*Abbreviations*: *BMI* Body mass index, *PB* Preterm birth

As presented in Table 2, BaP-DNA adducts were higher among PB (3.56 ± 1.46 ug/gDNA) than term birth (3.21 ± 0.83 ug/gDNA) with boundary significance (*p* = 0.058). We also compared the dietary intake levels of vitamins, folate, and carotene between PB and term births by t-tests. Higher levels of dietary VC, VA, VE, folate and carotene intake were observed in women delivered term births compared to those in PB group during preconception and three trimesters (post-conception), respectively. The statistical significances of these differences were observed for dietary VA, VE, folate, and carotene during preconception (*p* = 0.0059, 0.035, 0.043, and 0.012), VA and carotene during the first trimester (*p* = 0.044 and 0.034), and carotene only during the third trimester (*p* = 0.048). Additionally, among ever users of supplementation during preconception, women delivered term births (*N* = 23) took more times of supplements (*p* = 0.009) than those who delivered PB (*N* = 23), however, we found no differences between cases and controls during pregnancy, and three trimesters respectively.

**Table 2:** Differences of maternal BaP-DNA adducts, dietary vitamins, folate, and carotene levels between PB and term births

Characteristics	Term births (*N* = 82)		PBs (*N* = 83)		*P*^a^
	Mean	SD	Mean	SD	
BaP-DNA adducts (µg/gDNA)	3.21	0.83	3.56	1.46	*0.058*
Vitamin C (mg)^b^					
Preconception	105.4	37.5	95.0	32.4	*0.08*
1^st^ trimester	104.7	36.1	97.7	31.8	*0.21*
2^nd^ trimester	105.1	36.0	98.5	33.2	*0.25*
3^rd^ trimester	104.1	40.0	98.2	32.0	*0.30*
Vitamin A (µg RE)^b^					
Preconception	579.2	130.6	511.0	160.9	*0.0059*
1^st^ trimester	587.3	141.3	536.3	162.3	*0.044*
2^nd^ trimester	589.0	142.4	541.2	190.0	*0.091*
3^rd^ trimester	585.5	150.7	535.5	164.7	*0.057*
Vitamin E (mg)^b^					
Preconception	20.9	10.7	17.3	9.7	*0.035*
1^st^ trimester	20.8	10.6	17.7	9.7	*0.062*
2^nd^ trimester	21.0	10.6	17.8	15.6	*0.052*
3^rd^ trimester	20.5	9.2	17.8	9.7	*0.07*
Folate (µg)^b^					
Preconception	189.9	80.4	162.8	76.7	*0.043*
1^st^ trimester	189.9	78.4	165.7	74.7	*0.058*
2^nd^ trimester	191.1	77.8	169.5	82.1	*0.10*
3^rd^ trimester	186.4	68.2	168.2	73.8	*0.12*
Carotene (µg)^b^					
Preconception	3037.9	728.2	2689.8	897.0	*0.012*
1^st^ trimester	3008.4	738.0	2727.4	844.4	*0.034*
2^nd^ trimester	3035.1	738.9	2795.1	1045.6	*0.12*
3^rd^ trimester	3029.3	765.4	2762.9	851.8	*0.048*
Supplement intake times among ever users, N (term, PB)					
Preconception & during pregnancy (67, 66)	561.3	272.1	515.9	250.1	*0.32*
Preconception (23,23)	64.5	24.5	124.2	98.4	*0.009*
1^st^ trimester (60, 59)	297.6	85.5	297.1	71.9	*0.97*
2^nd^ trimester (46, 35)	233.0	115.6	213.5	115.8	*0.45*
3^rd^ trimester (37, 31)	203.9	106.5	199.9	101.6	*0.87*

^a^Calculated by t-test

*Abbreviations PB* Preterm birth, *BaP* benzo[a]Pyrene, *SD* Standard deviation

After adjustment for potential confounding factors in association analyses (Table 3 and supplementary table 1), an increased risk of PB and a decrease in gestational age were observed as per interquartile increase in maternal BaP-DNA adduct level (OR = 1.27, 95%CI 0.95–1.67; β = -0.23, *p* = 0.23). Compared to lower level of maternal adducts (below mean level: ≤ 3.21 µg/gDNA), high level of adducts (above mean level: > 3.21 µg/gDNA) was associated with the risk of PB, as well as a decrease in gestation age (OR = 2.05, 95%CI 1.05–4.01; β = -0.56, *p* = 0.19). For dietary intake, compared to low levels of intake (below mean level), high levels (above mean level) of VC, VA, VE, folate, and carotene were associated with a 43%—68% reduced risk of PB during different pregnancy windows, though some associations “during pregnancy only” or “preconception & during pregnancy” showed non-significant *p* values. After additional adjusted for supplementation status, slight greater associations were observed (Table 3). Positive associations were observed through additional analyses between dietary VC, VA, VE, folate, and carotene and gestational age (supplementary table 1). However, no significant association was found between dietary nutrients and maternal adducts levels (supplementary table 2).

**Table 3:** Associations between maternal BaP-DNA adducts and risk of PB, and between dietary vitamins, folate, carotene intake and risk of PB by different pregnancy windows

Characteristics & time windows	OR^a^	95%CI	OR^b^	95%CI
Maternal BaP-DNA adducts (µg/gDNA)				
Per interquartile increase	1.27	0.95, 1.67	1.28	0.95, 1.72
> 3.21 compared to ≤ 3.21	2.05	1.05, 4.01	2.09	1.06, 4.11
Dietary intake > mean levels compared to ≤ mean levels				
Vitamin C (mg)				
Preconception & during pregnancy	0.43	0.21, 0.87	0.39	0.19, 0.80
During pregnancy only	0.55	0.27, 1.10	0.48	0.24, 0.99
Preconception only	0.39	0.18, 0.84	0.34	0.16, 0.76
Vitamin A (µg RE)				
Preconception & during pregnancy	0.47	0.23, 0.96	0.43	0.21, 0.89
During pregnancy only	0.56	0.28, 1.14	0.52	0.25, 1.07
Preconception only	0.39	0.19, 0.81	0.34	0.16, 0.71
Vitamin E (mg)				
Preconception & during pregnancy	0.49	0.23, 1.04	0.45	0.21, 0.97
During pregnancy only	0.57	0.27, 1.19	0.54	0.26, 1.13
Preconception only	0.38	0.19, 0.80	0.35	0.16, 0.73
Folate (µg)				
Preconception & during pregnancy	0.32	0.16, 0.67	0.30	0.14, 0.63
During pregnancy only	0.40	0.20, 0.81	0.38	0.19, 0.78
Preconception only	0.38	0.18, 0.79	0.35	0.16, 0.74
Carotene (µg)				
Preconception & during pregnancy	0.43	0.21, 0.89	0.37	0.17, 0.78
During pregnancy only	0.50	0.24, 1.01	0.43	0.21, 0.90
Preconception only	0.39	0.19, 0.81	0.33	0.16, 0.71

^a^Adjusted for maternal age, education, family income per month, maternal BMI, passive smoking, parity, C-section, newborn gender, activity, and employment during pregnancy

^b^Additional adjusted for supplementation status (ever, never)

*Abbreviations*: *PB* Preterm birth, *BaP* Benzo[a]Pyrene, *OR* Odds Ratio, *CI* Confidence interval

Table 4 presented the associations between BaP-DNA adducts and risk of PB stratified by mean levels of dietary intakes. During “preconception only”, in groups of low levels of dietary intakes of all three vitamins, folate, and carotene, women who had above 3.21 µg of adducts had significantly increased risks of delivering PB babies, compared to those who had lower than 3.21 µg of adducts (OR = 4.83, 95%CI 1.31–17.80 for VC, OR = 2.94, 95%CI 1.08–7.98 for VA, OR = 4.88, 95%CI 1.75–13.65 for VE, OR = 2.79, 95%CI 1.16–6.72 for folate, OR = 2.64, 95%CI 1.01–6.92 for carotene). Similarly, during “pregnancy only” and “preconception & during pregnancy”, only in groups of low levels of dietary VE and folate intake, we estimated approximately 2–threefold times increase in risk of PB. As per quartile increase in BaP-DNA adducts in low levels of VE and folate intake, there were significantly 46%-69% increased risks of PB observed during pregnancy and/or preconception. However, there was no association found in groups of high levels of dietary intake for all vitamins, folate, and carotene. Tests for interaction terms between adducts and dietary intakes showed statistically non-significant (*P* > 0.05). Similar patterns were observed after additional adjustment of supplementation (Table 5).

**Table 4:** Associations between maternal BaP-DNA adducts and risk of PB stratified by mean levels of dietary vitamins, folate, and carotene during different pregnancy windows

BaP-DNA adducts (µg/gDNA)	Preconception & During pregnancy				During pregnancy only				Preconception only			
	≤ Mean levels		> Mean levels		≤ Mean levels		> Mean levels		≤ Mean levels		> Mean levels	
	OR^a^	95%CI	OR^a^	95%CI	OR^a^	95%CI	OR^a^	95%CI	OR^a^	95%CI	OR^a^	95%CI
*Vitamin C*												
Per interquartile increase	1.33	0.89, 1.99	1.22	0.82, 1.81	1.36	0.82, 2.27	1.29	0.77, 2.15	1.49	0.90, 2.46	1.32	0.85, 2.05
> 3.21 compared to ≤ 3.21	2.39	0.93, 6.18	2.14	0.85, 5.40	2.10	0.69, 6.45	2.05	0.68, 6.19	4.83	1.31, 17.80	1.95	0.75, 5.04
*Vitamin A*												
Per interquartile increase	1.20	0.79, 1.81	1.37	0.83, 2.27	1.25	0.82, 1.90	1.42	0.87, 2.32	1.33	0.87, 2.03	1.21	0.73, 2.01
> 3.21 compared to ≤ 3.21	2.24	0.85, 5.93	2.06	0.70, 6.12	2.25	0.84, 6.05	2.56	0.89, 7.38	2.94	1.08, 7.98	1.55	0.48, 4.97
*Vitamin E*												
Per interquartile increase	1.37	0.95, 1.97	1.27	0.69, 2.35	1.46	1.01, 2.12	1.17	0.64, 2.15	1.62	1.05, 2.50	1.36	0.79, 2.32
> 3.21 compared to ≤ 3.21	2.81	1.27, 6.68	1.09	0.27, 4.33	3.03	1.31, 7.03	0.98	0.25, 3.87	4.88	1.75, 13.65	1.28	0.40, 4.10
*Folate*												
Per interquartile increase	1.55	1.03, 2.34	1.42	0.78, 2.59	1.69	1.11, 2.57	1.26	0.70, 2.27	1.41	0.95, 2.10	1.67	0.90, 3.09
> 3.21 compared to ≤ 3.21	3.42	1.37, 8.58	1.54	0.40, 5.94	3.78	1.49, 9.60	1.19	0.31, 4.53	2.79	1.16, 6.72	2.79	0.66, 11.86
*Carotene*												
Per interquartile increase	1.22	0.82, 1.81	1.35	0.80, 2.27	1.26	0.84, 1.89	1.36	0.82, 2.27	1.30	0.86, 1.98	1.23	0.74, 2.05
> 3.21 compared to ≤ 3.21	2.14	0.85, 5.40	2.08	0.65, 6.89	2.42	0.94, 6.24	2.10	0.69, 6.45	2.64	1.01, 6.92	1.57	0.47, 5.28

^a^Adjusted for maternal age, education, family income per month, maternal BMI, passive smoking, parity, C-section, newborn gender, activity, and employment during pregnancy

*Abbreviations*: *PB* Preterm birth, *BaP* Benzo[a]Pyrene, *OR* Odds Ratio, *CI* Confidence interval

**Table 5:** Associations between maternal BaP-DNA adducts and risk of PB stratified by mean levels of dietary vitamins, folate, and carotene during different pregnancy windows with additional adjustment for supplementation

BaP-DNA adducts (µg/gDNA)	Preconception & During pregnancy				During pregnancy only				Preconception only			
	≤ Mean levels		> Mean levels		≤ Mean levels		> Mean levels		≤ Mean levels		> Mean levels	
	OR^a^	95%CI	OR^a^	95%CI	OR^a^	95%CI	OR^a^	95%CI	OR^a^	95%CI	OR^a^	95%CI
*Vitamin C*												
Per interquartile increase	1.32	0.88, 1.98	1.46	0.84, 2.55	1.31	0.87, 1.98	1.38	0.82, 2.34	1.52	0.92, 2.52	1.34	0.86, 2.10
> 3.21 compared to ≤ 3.21	2.35	0.90, 6.13	2.44	0.73, 8.20	2.28	0.88, 5.90	2.13	0.68, 6.65	5.21	1.38, 19.75	1.99	0.76, 5.23
*Vitamin A*												
Per interquartile increase	1.13	0.74, 1.73	1.40	0.84, 2.34	1.18	0.76, 1.81	1.45	0.87, 2.39	1.24	0.80, 1.92	1.23	0.74, 2.06
> 3.21 compared to ≤ 3.21	2.11	0.78, 5.73	2.12	0.70, 6.39	2.12	0.77, 5.85	2.63	0.89, 7.71	2.72	0.97, 7.62	1.58	0.49, 5.11
*Vitamin E*												
Per interquartile increase	1.36	0.94, 1.97	1.51	0.78, 2.91	1.45	1.00, 2.10	1.34	0.71, 2.55	1.59	1.03, 2.46	1.55	0.87, 2.77
> 3.21 compared to ≤ 3.21	2.97	1.27, 6.94	1.38	0.32, 5.92	3.07	1.31, 7.23	1.19	0.29, 4.97	4.92	1.73, 14.01	1.47	0.44, 4.88
*Folate*												
Per interquartile increase	1.52	1.00, 2.30	1.66	0.86, 3.18	1.66	1.09, 2.53	1.41	0.75, 2.63	1.37	0.92, 2.06	1.67	0.97, 3.63
> 3.21 compared to ≤ 3.21	3.36	1.32, 8.57	1.79	0.45, 7.20	3.71	1.45, 9.50	1.35	0.34, 5.34	2.70	1.10, 6.62	3.17	0.72, 14.02
*Carotene*												
Per interquartile increase	1.16	0.77, 1.76	1.36	0.80, 2.32	1.20	0.79, 1.83	1.37	0.81, 2.31	1.21	0.79, 1.86	1.25	0.74, 2.10
> 3.21 compared to ≤ 3.21	1.99	0.77, 5.15	2.10	0.63, 7.05	2.27	0.86, 6.06	2.12	0.68, 6.59	2.34	0.87, 6.29	1.60	0.47, 5.46

^a^Adjusted for maternal age, education, family income per month, maternal BMI, passive smoking, parity, C-section, newborn gender, activity, employment during pregnancy, and supplementation status

*Abbreviations*: *PB* Preterm birth, *BaP* Benzo[a]Pyrene, *OR* Odds Ratio, *CI* Confidence interval

## Discussion

Our study suggested that maternal exposure to elevated levels of PAHs, indicated by BaP-DNA adducts from maternal whole blood, were associated with an increased risk of PB, and the increased risk varied by dietary vitamins, folate, and carotene intake levels during preconception and pregnancy. Especially, the significant increased risks were consistently seen for all nutrients during preconception-12 months before pregnancy.

Our study is the first one that observe an increased risk of PB associated with high level of maternal BaP-DNA adducts, measured by an improved LC–MS/MS method published in 2019 [29] with small amount of 10 µg DNA. Consistent with previous evidence that indirectly revealed the relationship between BaP-DNA adducts and PB. Siter et al. found significantly elevated levels of BaP in placenta from preterm deliveries (*N* = 22) compared with terms (*N* = 20) residing near Superfund sites, and increased DNA adducts by ^32^P-postlabeling method in subjects residing in zip codes which contain Superfund sites compared to those who do not [12]. Similarly, other studies have reported an increased risk of PB among women with higher levels of PAHs exposure, estimated based on personal measures of atmospheric PAH exposure by gas chromatograph/HPLC [9, 12, 13], or by air monitoring data assignment [7]. The main reason for not applying the DNA adducts as an indicator of PAH exposure in these earlier studies is that the regular LC–MS/MS method will require very large amounts of DNA [12], even the HPLC method for measuring personal blood or placenta PAHs levels also require as much as 100 µg DNA [9, 14]. Among several methods for adducts analysis, including ^32^P-postlabeling, immunoassay, and LC–MS/MS, an ultrasensitive LC–MS/MS used in recent study has been approved as the best analytical method [32], since it is based on the effective purification and enrichment of BPDE-dG adducts in human samples followed by solvent extraction to efficiently enrich the adducts, and by adding as an internal standard to improve the better recovery [29]. BaP-DNA adducts have been identified as a representative biomarker of overall PAH exposure, including PAHs from tobacco smoke, air pollution from atmosphere or cooking fuel, as well as the diet [33]. With 3–4 months half-life, BaP-DNA adducts levels reflect fluctuations in the timing, duration, intensity, and individual variations in exposure, absorption, metabolic activation, and DNA repair of maternal and fetal exposure to PAHs [20].

We also found the protect effects of high levels of dietary vitamins, folate, and carotene intakes on risk of PB, as well as the levels of above mean dietary intakes may modify the association between BaP-DNA adducts and PB. Duarte-Salles et al. reported dietary BaP level were associated with reductions in birth weight and length, and increased risk of infants with small for gestational age among women with low dietary VC [21]. Another study found that VC and VE supplementation appears to be associated with reduction in placental abruption and preterm birth among smokers [25]. However, the targeted chemical compounds from cigarette/tobacco such as PAHs [34] was not examined in this study. Not only we reached the same conclusion among low dietary VC and VE group, but we additionally observed similar results among groups of low dietary VA, folate and carotene. Biologically, high level of VC has been identified to decrease the frequency of genomic translocations, micronuclei, DNA strand breakage, and oxidative damage, but modulate of DNA repair, when exposed to PAHs in polluted air [35]. In an animal study, DNA adduct formation in rat glial tumor cells was decreased by VC [36]. Moreover, the effects of VA, VE, carotene, and folate on decreases of adducts formation has also been reported. Specifically, VE prevented cells from BaP-induced cell cycle arrest and growth inhibition, significantly suppressed BaP-induced reactive oxygen species levels, and decreased BaP-DNA adducts [37]. VA and beta-carotene could decrease the level of BaP-DNA adducts in hamster tracheal epithelial cells with 18% and 40% by enhancing DNA-repair activities [22]. A clinical trial reported a significant reduction in BaP-DNA adducts in female smokers with vitamin treatment [38]. Meanwhile, folate could restrain BaP-induced cyclinD1 protein expression, which helps cells return to their normal cell cycle [39]. An inverse association between dietary folate intake and lung adduct levels was observed among individual, and the combination of low folate intake and impaired folate metabolic polymorphisms may be implicated in DNA damage in target lung tissue [40]. However, most studies concerning the effect of vitamins, carotene, and folate on DNA adducts are focused on tumor initiation, promotion and progression. In women delivered PB, the levels of placental gene expression of various phase I and phase II drug metabolizing enzymes-Ah receptor, *NRF2*, and *CYP1A1* were decreased [12]. *CYP1A1* has a role in the formation of Ah receptor ligands that help to maintain pregnancy and has been identified to be associated with the rate and occurrence of PB [41]. The decrease in *NRF2* expression in preterm placenta was consistent with the hypothesis that increased levels of BaP-induced ROS may have contributed to detectable and significant differences in PAHs accumulation [42]. Therefore, it is biologically plausible that dietary vitamins, carotene, and folate may attenuate the adverse effects of maternal PAHs exposure on PB.

Interestingly, the positive associations between adducts and PB risk among low intake of vitamins, carotene, and folate were observed consistently during preconception, indicating that earlier intakes of high level of these nutrients had a beneficial effect on reduction of PB risk when exposure to high level of PAHs during pregnancy. Though the available data indicate the importance of pregnant women's nutrition such as vitamin, mineral, and folate supplements prior to and during the first trimester of pregnancy [30, 43], no studies did explore their effects on associations between PAHs exposure and PB risk by different pregnancy windows including preconception. Future studies are needed to confirm and replicate the results, and to explore the underlying mechanism.

Our study firstly examined the association between BaP-DNA adducts and PB risk, as well as the effects of dietary vitamins, carotene, and folate on this association. A major strength is that we used an improved LC-MC/MC analytic method to detect the BaP-DNA adducts which is a direct estimate of overall airborne PAHs from several sources, whereas some previous studies used monitoring pollution data assignments as an indirect estimation of PAHs exposure. Though statistical power was limited for stratified analysis by preterm subtypes and the significant interactions were not observed, our study included a relatively large sample size (*N* = 165) compared to earlier studies. Due to the small sample size, chance findings cannot be ruled out, and the large variance of the estimator might make association toward the null. The distributions of selected characteristics were different between the study population and the excluded population in the entire birth cohort (supplementary table 3) [44], which raised a concern about representativeness of the entire birth cohort and generalizability of the study results to other populations. Future studies are needed to replicate our results in different populations with greater statistical power. Detailed information on potential confounding factors were collected and controlled for in analyses. Birth outcomes and maternal complications during pregnancy were obtained from medical records, which minimized potential disease misclassification. Because information on dietary was collected through in-person interview at delivery, there was potential for recall bias. However, the relationships between BaP-DNA adducts, dietary nutrients intakes, and risk of PB have not been well-established, and were unlikely to be known by the general public. Therefore, if there was any recall bias it was likely to be non-differential and resulted in underestimation of the observed associations. Because of the limited sample size for some pregnancy periods (preconception or during pregnancy) among supplementation users, the stratified analysis by supplementation frequency on the association were not performed in our analyses. However, we found there was no different between PB and terms for supplementation status (ever/never) (Table 1) and frequency during pregnancy (Table 2). As additionally adjusting it as a potential confounding factor in the model, we compared the results with/without supplementation and reached the same conclusion (Tables 3, 4 and 5). Future studies are necessary to identify the effect of supplementation on PAHs exposure in relation to PB.

## Conclusions

Our study supports the hypothesis that high level of maternal PAHs exposure was significantly associated with increased risk of PB, and provides the first evidence that dietary vitamins, carotene, and folate intake levels may modify this association during different pregnancy windows. Specifically, greater increased PB risk was consistently observed among women with low dietary nutrients intakes prior to and during pregnancy. The findings from our study have important public health implications and are relevant to identify policy recommendation for environment management regarding pregnant women and newborns. It may facilitate acceptance of taking more nutrients/supplements during both pre- and post-conception.

## Supplementary Information

**Additional file 1: Supplementary Table 1.** Associations between maternal BaP-DNA adducts, dietary vitamins, folate, carotene intake preconception & during pregnancy and gestational age at delivery. **Supplementary Table 2.** Associations between dietary vitamins, folate, carotene intake preconception & during pregnancy and maternal BaP-DNA adducts. **Supplementary Table 3.** Distributions of selected characteristics between the study subjects in and not in current study.

## Data Availability

The datasets used and/or analyzed during the current study are available from the corresponding author on reasonable request.

## Abbreviations

PAHs: Polycyclic aromatic hydrocarbons
PB: Preterm birth
BaP: Benzo[a]pyrene
WHO: World Health Organization
BPDE-dG: Benzo[a]pyrenediol epoxide-dG
HPLC: High-performance liquid chromatrography
VA: Vitamin A
VC: Vitamin C
VE: Vitamin E
BMI: Body mass index
CI: Confidence interval
OR: Odds Ratio
