# Correction: Effects of Benzo [a]pyrene-DNA adducts, dietary vitamins, folate, and carotene intakes on preterm birth: a nested case–control study from the birth cohort in China

**DOI:** 10.1186/s12940-022-00867-7

**Published:** 2022-05-24

**Authors:** Nan Zhao, Weiwei Wu, Shiwei Cui, Haibin Li, Yongliang Feng, Ling Guo, Yawei Zhang, Suping Wang

**Affiliations:** 1 Medical Research Center/State Key Laboratory of Complex Severe and Rare Diseases, Peking Union Medical College Hospital, Chinese Academy of Medical Sciences and Peking Union Medical College, Beijing, China; 2 Department of Epidemiology, School of Public Health, Shanxi Medical University, Taiyuan, Shanxi China; 3 Chinese Center for Disease Control and Prevention, National Institute for Occupational Health and Poison Control, Beijing, China; 4 Institute of Urban Safety and Environmental Science, Beijing Academy of Science and Technology, Beijing, China; 5 National Cancer Center/National Clinical Research Center for Cancer/Cancer Hospital, Chinese Academy of Medical Sciences and Peking Union Medical College, Beijing, China

**Correction: Environ Health 21, 48 (2022)**

**https://doi.org/10.1186/s12940-022-00859-7**

Following the publication of the original article [1], the author reported that the last author, Suping Wang, should not be affiliated with affiliation 3. The correct list and its affiliation is presented above.
