# Supplementary material for: Effects of Benzo[a]pyrene-DNA adducts, dietary vitamins, folate, and carotene intakes on preterm birth: a nested case–control study from the birth cohort in China
Source: Environ Health. 2022 May 6;21:48. doi: 10.1186/s12940-022-00859-7 (PMC9074263; doi:10.1186/s12940-022-00859-7)
Supplement: Supplementary file 1 — Additional file 1: Supplementary Table 1. Associations between maternal BaP-DNA adducts, dietary vitamins, folate, carotene intake preconception & during pregnancy and gestational age at delivery. Supplementary Table 2. Associations between dietary vitamins, folate, carotene intake preconception & during pregnancy and maternal BaP-DNA adducts. Supplementary Table 3. Distributions of selected characteristics between the study subjects in and not in current study. [file 12940_2022_859_MOESM1_ESM.docx]

| Supplementary Table 1: Associations between maternal BaP-DNA adducts, dietary vitamins, folate, carotene intake preconception & during pregnancy and gestational age at delivery | | | | |
| --- | --- | --- | --- | --- |
| Characteristics | | Gestational age | | |
|  |  | β^a^ | 95%CI | *P* |
| Maternal BaP-DNA adducts (µg/gDNA) | |  |  |  |
|  | Per interquartile increase | -0.23 | -0.60, 0.15 | *0.23* |
|  | >3.21 compared to ≤3.21 | -0.56 | -1.41, 0.28 | *0.19* |
| Dietary intake >mean levels compared to ≤ mean levels | | | | |
|  | Vitamin C (mg) | 1.24 | 0.35, 2.12 | *0.0065* |
|  | Vitamin A (µg RE) | 0.93 | 0.02, 1.84 | *0.046* |
|  | Vitamin E (mg) | 1.03 | 0.08, 1.98 | *0.033* |
|  | Folate (µg) | 1.14 | 0.25, 2.03 | *0.013* |
|  | Carotene (µg) | 0.97 | 0.06, 1.89 | *0.038* |
| ^a^ Adjusted for maternal age, education, family income per month, maternal BMI, passive smoking, parity, C-section, newborn gender, activity, and employment during pregnancy, and supplementation status (ever, never)  Abbreviations: BaP, Benzo(a)Pyrene | | | | |

| Supplementary Table 2: Associations between dietary vitamins, folate, carotene intake preconception & during pregnancy and maternal BaP-DNA adducts | | | | | | | |
| --- | --- | --- | --- | --- | --- | --- | --- |
| Characteristics | | Maternal BaP-DNA adducts (µg/gDNA) | | | | | |
|  |  | Per interquartile increase  (Linear regression) | | |  | >3.21 compared to ≤3.21  (Logistic regression) | |
|  |  | β^a^ | 95%CI | *P* |  | OR^a^ | 95%CI |
| Dietary intake >mean levels compared to ≤ mean levels | | | | | | | |
|  | Vitamin C (mg) | 0.28 | -0.11 0.66 | *0.16* |  | 1.24 | 0.63, 2.45 |
|  | Vitamin A (µg RE) | -0.07 | -0.46 0.33 | *0.73* |  | 1.06 | 0.53, 2.13 |
|  | Vitamin E (mg) | 0.28 | -0.13, 0.69 | *0.17* |  | 1.63 | 0.78, 3.39 |
|  | Folate (µg) | 0.31 | -0.08, 0.70 | *0.11* |  | 1.47 | 0.74, 2.92 |
|  | Carotene (µg) | 0.005 | -0.39, 0.40 | *0.98* |  | 0.96 | 0.48, 1.93 |
| ^a^ Adjusted for maternal age, education, family income per month, maternal BMI, passive smoking, parity, C-section, newborn gender, activity, and employment during pregnancy, and supplementation status (ever, never)  Abbreviations: BaP, Benzo(a)Pyrene | | | | | | | |

| Supplementary Table 3: Distributions of Selected Characteristics Between the Study Subjects in and not in Current Study | | | | | | | | | |
| --- | --- | --- | --- | --- | --- | --- | --- | --- | --- |
| Characteristics | | Subjects in current study (N=165) | | |  | Subjects not in current study (N=3001) | | | *P* ^a^ |
|  |  | N | | % |  | N | | % |  |
| Preterm | |  | |  |  |  | |  |  |
|  | No | 82 | | 49.7 |  | 2717 | | 90.5 | <.001 |
|  | Yes | 83 | | 50.3 |  | 284 | | 9.5 |  |
| Maternal age (years) | |  | |  |  |  | |  |  |
|  | <30 | 106 | | 64.2 |  | 1768 | | 58.9 | 0.17 |
|  | ≥30 | 59 | | 35.8 |  | 1233 | | 41.1 |  |
| Highest education level | |  | |  |  |  | |  |  |
|  | < College | 71 | | 43.0 |  | 975 | | 32.5 | 0.005 |
|  | ≥ College | 94 | | 57.0 |  | 2026 | | 67.5 |  |
| Family monthly income (RMB per capita) | | | | |  |  | |  |  |
|  | <3,000 | 79 | | 47.9 |  | 1649 | | 54.9 | 0.076 |
|  | ≥3,000 | 86 | | 52.1 |  | 1352 | | 45.1 |  |
| Employment during pregnancy | | | |  |  |  | |  |  |
|  | No | 101 | | 61.2 |  | 1406 | | 46.9 | <.001 |
|  | Yes | 64 | | 38.8 |  | 1595 | | 53.1 |  |
| Pre-pregnancy BMI ^b^ | |  | |  |  |  | |  |  |
|  | ≤18.5 | 29 | | 17.6 |  | 408 | | 13.6 | 0.30 |
|  | 18.5-24.0 | 110 | | 66.7 |  | 2042 | | 68.0 |  |
|  | ≥24.0 | 26 | | 15.8 |  | 551 | | 18.4 |  |
| Parity | |  | |  |  |  | |  |  |
|  | Primiparous | 108 | | 65.5 |  | 1483 | | 49.4 | <.001 |
|  | Multiparous | 57 | | 35.6 |  | 1518 | | 50.6 |  |
| C-section | |  | |  |  |  | |  |  |
|  | No | 92 | | 55.8 |  | 1886 | | 62.8 | 0.067 |
|  | Yes | 73 | | 44.2 |  | 1115 | | 37.2 |  |
| Gender | |  | |  |  |  | |  |  |
|  | No | 76 | | 46.1 |  | 1419 | | 47.3 | 0.76 |
|  | Yes | 89 | | 53.9 |  | 1582 | | 52.7 |  |
| Passive smoking during pregnancy | | | |  |  |  | |  |  |
|  | No | 145 | | 87.9 |  | 2617 | | 87.2 | 0.80 |
|  | Yes | 20 | | 12.1 |  | 384 | | 12.8 |  |
| Activities during pregnancy | | | |  |  |  | |  |  |
|  | No | 31 | | 18.8 |  | 314 | | 10.5 | <.001 |
|  | Yes | 134 | | 81.2 |  | 2687 | | 89.5 |  |
| Supplement intake | |  | |  |  |  | |  |  |
|  | Never | 32 | | 19.4 |  | 332 | | 11.1 | 0.001 |
|  | Ever | 133 | | 80.6 |  | 2669 | | 88.9 |  |
|  | | Mean | SD | |  | | Mean | SD | *P* ^a^ |
| Vitamin C (mg) | | 100.6 | 34.2 | |  | | 102.4 | 32.4 | 0.52 |
| Vitamin A (µg RE) | | 560.7 | 150.3 | |  | | 539.2 | 166.9 | 0.13 |
| Vitamin E (mg) | | 19.2 | 9.8 | |  | | 18.1 | 11.4 | 0.21 |
| Folate (µg) | | 179.0 | 76.1 | |  | | 175.8 | 93.0 | 0.63 |
| Carotene (µg) | | 2902.6 | 815.7 | |  | | 2811.7 | 905.5 | 0.24 |
| ^a^ Calculated by t-test for dietary nutrients, by Chi-square analysis for other characters  ^b^ Weight (kg) / height (m)^2^  Abbreviations: BMI, body mass index; PB, preterm birth; SD, standard deviation | | | | | | | | | |
